# Comparing the Effectiveness of Different Types of Orthoses in Preventing Diabetic Foot Ulcer Recurrence: A Systematic Review of Randomized Clinical Trials

**DOI:** 10.1002/jfa2.70192

**Published:** 2026-08-01

**Authors:** Behnam Amini, Hanieh Salehi‐Pourmehr, Sevil Ghaffarzadehrad

**Affiliations:** ^1^ Health Services Management Department Faculty of Management and Medical Information Tabriz University of Medical Sciences Tabriz Iran; ^2^ Research Center for Evidence‐Based Medicine Iranian EBM Centre: A JBI Centre of Excellence Faculty of Medicine Tabriz University of Medical Sciences Tabriz Iran; ^3^ Endocrine Research Center Tabriz University of Medical Sciences Tabriz Iran

**Keywords:** diabetes, foot ulcer, insoles, orthoses, peripheral neuropathy, therapeutic footwear

## Abstract

**Introduction:**

Therapeutic orthoses, such as medical shoes and custom insoles, are used to prevent diabetic foot ulcer (DFU) recurrence. This systematic review with narrative synthesis aimed to evaluate the effectiveness of orthoses in preventing DFU recurrence compared to standard care.

**Methods:**

We followed PRISMA guidelines for a systematic review. Searches were conducted in PubMed, Cochrane, and Web of Science. RCTs on orthoses for preventing DFU recurrence were included. Risk of bias was assessed using Cochrane tools. Due to heterogeneity, narrative synthesis was used.

**Results:**

An initial search of 4166 articles identified ten randomized controlled trials (RCTs) that met the inclusion criteria and were included in this review, focusing on patients living with diabetes at risk of DFU recurrence compared to usual care. Narrative synthesis of the 10 RCTs, involving 1686 participants, showed that custom orthoses reduced recurrence in several studies (e.g., Bus 2013 reported 9.9% recurrence vs. 25% in controls), though the evidence was heterogeneous due to variability in orthosis types and follow‐up periods.

**Conclusion:**

Orthoses can prevent diabetic foot ulcer recurrence, and we recommend their use where feasible. Further research is needed to optimize their clinical application.

## 1 Introduction

Diabetes is a chronic condition that affects the body's ability to regulate blood sugar levels, leading to various health complications over time [1]. These complications can include cardiovascular diseases, kidney damage, and nerve damage, which can result in neuropathy. Additionally, diabetes can result in vision problems, such as diabetic retinopathy, and increase the risk of infections due to a weakened immune system [2]. Maintaining proper blood sugar levels is crucial to prevent or minimize these serious health issues. Diabetic foot ulcer is considered one of the most prevalent and critical complications of diabetes mellitus, affecting approximately 25% of individuals living with diabetes during their lifetime [3]. It is caused by a combination of factors, including poor circulation, nerve damage, and infection. The etiology of diabetic foot ulceration involves peripheral neuropathy, which results in a loss of sensation in the feet, and peripheral vascular disease, which results in reduced blood flow [4]. Impairment of wound prevention and an elevated possibility of infection are often observed in such conditions, making recovery more difficult and prolonged. Treatment of diabetic foot ulcers can be difficult, and there are many complications associated with diabetic foot ulcers, including infection, osteomyelitis, and amputation [5]. In severe cases, untreated ulcers can lead to gangrene, necessitating surgical intervention or even amputation to prevent the spread of infection [6]. In addition to causing significant patient discomfort and reduced quality of life, these complications can also increase the cost of healthcare. Therefore, recognizing the signs and symptoms of diabetic foot ulceration early and taking prompt action to prevent serious complications is of high importance.

Orthoses, or custom‐made shoe inserts, can be an effective treatment for diabetic foot ulceration [7]. Orthoses are specially designed foot supports that help to redistribute pressure, improve foot function, and reduce the risk of ulcers by providing cushioning and stability. Studies have shown that approximately 15% of people with diabetes will develop a foot ulcer during their lifetime [8]. These ulcers can lead to serious complications if not treated properly, making orthoses a crucial intervention in preventing further issues. They work by redistributing pressure away from the ulcer site, preventing ulcer recurrence and preventing further damage [9]. Orthoses can also help improve balance and stability, reducing the risk of falls and further injury [10]. It is important to consult with a healthcare professional, such as a podiatrist, to determine the appropriate type of orthosis for each individual case. Proper management of diabetes mellitus and prevention of diabetic foot ulceration, such as wearing appropriate footwear and seeking prompt medical attention, are crucial to preventing diabetic foot ulceration [11, 12].

Although some studies have reported beneficial effects, the overall effectiveness of custom‐made orthoses in preventing DFU recurrence in individuals living with diabetes remains uncertain. Given limited healthcare resources, this systematic review with narrative synthesis aimed to assess the effectiveness of orthotic devices compared to standard care in preventing DFU recurrence.

Diabetes is a chronic condition that impairs the body's ability to regulate blood sugar, leading to complications such as cardiovascular diseases, kidney damage, and neuropathy [1]. Additional risks include vision impairment, such as diabetic retinopathy, and increased susceptibility to infections due to a weakened immune system [2]. Maintaining proper blood sugar levels is critical to minimizing these serious health issues. Diabetic foot ulcers (DFUs) are among the most prevalent and severe complications of diabetes mellitus, affecting approximately 25% of individuals living with diabetes during their lifetime [3]. These ulcers result from a combination of peripheral neuropathy, which causes loss of sensation in the feet, and peripheral vascular disease, which reduces blood flow [4]. These conditions increase the risk of ulcer recurrence, infection, and complications such as osteomyelitis or amputation [5]. In severe cases, untreated ulcers may lead to gangrene, necessitating surgical intervention to prevent further complications [6]. DFUs cause significant discomfort, reduce quality of life, and increase healthcare costs, making early recognition and prevention of ulcer recurrence a priority.

Orthoses, such as custom‐made shoe inserts, are designed to redistribute pressure, enhance foot function, and reduce the risk of DFU recurrence by providing cushioning and stability [7]. Studies estimate that 15% of individuals with diabetes will develop a foot ulcer at some point, with recurrence being a major concern if not properly managed [8]. Orthoses work by offloading pressure from high‐risk areas, thereby reducing the likelihood of ulcer recurrence [9]. They also improve balance and stability, decreasing the risk of falls and further injury [10]. Consultation with a healthcare professional, such as a podiatrist, is essential to select the appropriate orthosis for each patient. Effective diabetes management, including the use of appropriate footwear and timely medical care, is crucial for preventing DFU recurrence [11, 12].

Despite reported benefits, the overall effectiveness of custom‐made orthoses in preventing DFU recurrence in individuals living with diabetes remains uncertain. Given limited healthcare resources, this systematic review with narrative synthesis aimed to assess the effectiveness of orthotic devices compared to standard care in preventing diabetic foot ulcer recurrence.

## 2 Methods

The present rapid systematic review was conducted based on the World Health Organization (WHO) guidelines for rapid reviews, and its results were reported according to the Preferred Reporting Items for Systematic Reviews (PRISMA) guidelines. Moreover, the study protocol was prospectively registered in the International Prospective Register of Systematic Reviews (PROSPERO) with the registration number CRD42025643175.

We used the PRISMA guide for systematic review with narrative synthesis. Due to high heterogeneity in orthotic type and follow‐up periods (I^2^ > 50% in primary analysis), meta‐analysis was abandoned and we used narrative synthesis.

### 2.1 Search Strategy

Briefly, this study was done to compare the efficacy of different types of orthoses (such as medical shoes, custom insoles, braces) in healing diabetic foot ulcers. The PICO strategy was used to establish the guiding question of this review.

P: Patients With Diabetes at Risk of DFU Recurrence

I: Different types of orthoses (e.g., custom insoles, therapeutic footwear, braces).

C: Standard Care (e.g., Routine Education, Standard Footwear)

O: ulcer recurrence Searches were exhaustively conducted on a systematic basis through PubMed, Cochrane, Cochrane Protocols and Trials, Scopus, Web of Science, Google Scholar, Irandoc, ISC, and Magiran with no time limitation up to 2024. The following keywords and medical subject headings (MeSH) were combined: “Foot Ulcer” OR “Diabetic Foot” OR “Wound Healing”) AND (“Foot Orthoses” OR “Braces” OR “Orthotic Devices”) AND (“Diabetes Mellitus” OR “Diabetes Mellitus, Type 2” OR ”Diabetes Mellitus, Type 1”). No limitations or restrictions were applied to the search strategy during the literature search process (Supporting Information S1).

### 2.2 Inclusion and Exclusion Criteria

**FIGURE 1:**
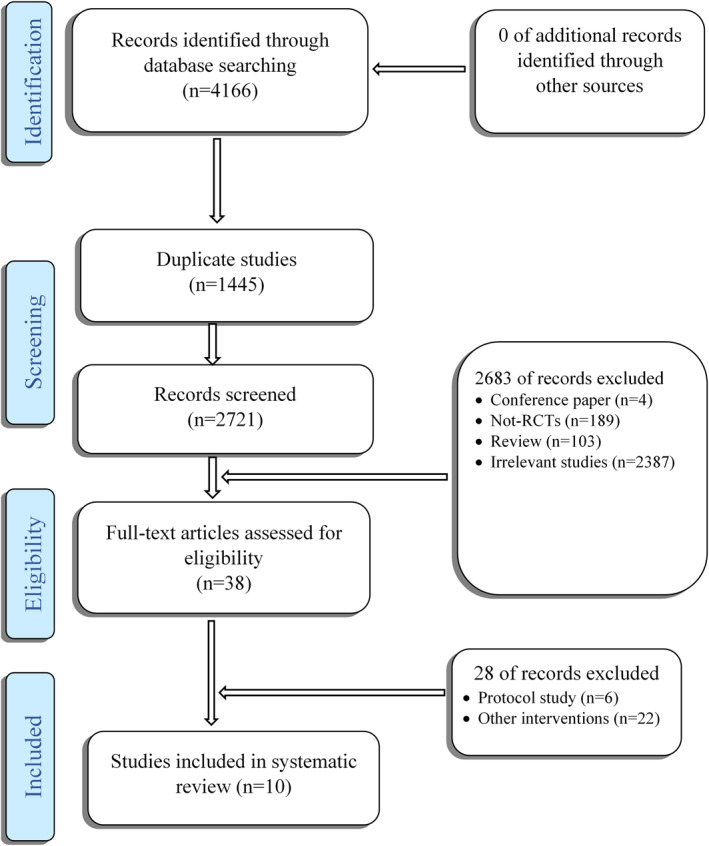
Flow diagram to summarize the search strategy.

All published randomized clinical trials (RCTs) on the efficacy of various types of orthoses in healing diabetic foot ulcers, regardless of the study time and language, were reviewed. All observational (cross‐sectional, case‐control, and cohort), animal studies, case reports, narrative reviews, systematic reviews, and letters to the editor were excluded. In this study, the primary outcome was ulcer recurrence at the end of intervention (Figure 1). Usual care in the studies included routine foot care education and the use of standard, non‐customized shoes, as reported in the included studies.

### 2.3 Study Selection

The titles and abstracts retrieved through the literature search were independently screened by two reviewers after ethics committee approval. Full texts of all potentially eligible studies were independently downloaded and evaluated by both reviewers. Any discrepancies in study selection were resolved through discussion with a third reviewer.

### 2.4 Data Extraction

A predesigned extraction form was used by two authors to independently screen the literature and extract the data. The extracted data included the first author's name, journal, year of publication, study setting, country of origin, and sample size, comparator, outcomes assessed, adverse events & safety, sample size, inclusion criteria, intervention description, baseline characteristics, statistical results, and other results. Two authors extracted data independently from the qualified articles. If the primary data had been reported graphically, we applied universal desktop ruler software to measure the numerical values from the graph.

### 2.5 Quality Assessment

Risk of bias was assessed by two authors independently, with the guidance of the Cochrane Collaboration Handbook [13]The following biases were assessed: random sequence generation (selection bias), allocation concealment (selection bias), blinding of participants and personnel (performance bias), blinding of outcome assessment (detection bias), incomplete outcome data (attrition bias), and selective reporting (reporting bias) in controlled trial studies. Additionally, the ROBINS‐I tool was used to evaluate biases in quasi‐experimental studies [14]. In the next step, judgments were reconciled and in case of disagreement, a third person was consulted. The bias of the studies was shown by Review Manager 5.3 software (RevMan; The Cochrane Collaboration, Oxford, UK).

## 3 Results

### 3.1 Study Selection

Figure 1 illustrates the PRISMA statement‐based search and screening process for the literature. When the initial search was conducted, 4166 articles were found. Incorporating inclusion/exclusion criteria resulted in the rejection of 4156 articles based on titles, abstracts, and full texts. An analysis of 10 qualified publications was eventually conducted [15, 16, 17, 18, 19, 20, 21, 22, 23, 24]. Study dates ranged from 1994 to 2024 for all included studies.

### 3.2 Study Characteristics

A total of 1686 participants were included across 10 studies published between 1995 and 2024. Of these, 887 participants were assigned to the intervention group (orthoses), while 799 participants were in the control group (usual care). Specifically, custom‐made optimized insoles were used in three studies [16, 17, 18], footwear was utilized in three studies [15, 19, 23], and four studies were used on orthoses [20, 21, 22, 24]. The length of treatments spanned from 3 months to 18 months. In Table 1, we provide detailed information for the included studies.

**TABLE 1:** Evidence table for randomized controlled trials (RCTs) Usual Care/Standard Care Description.

Reference, intervention categories and outcomes reported	Design, setting	Population, intervention (I), control (C) and follow‐up period	Population characteristics	Outcome findings Usual Care/Standard Care Description	
**Bus et al. (2013)** **Intervention categories:** Custom‐made footwear **Outcomes reported:** Loss of protective sensation Peripheral arterial status Foot deformity Regional mean peak pressures	**Study design:** RCT **Setting(s):** Netherlands.	**Population:** Patients with age > 18 years, confirmed type 1 or type 2 diabetes, loss of protective foot sensation, a healed plantar foot ulcer. **Exclusion criteria:** Bilateral amputation Use of walking aids that offload the foot Severe illness **Intervention (I):** Custom‐made footwear of which the offloading properties were improved and subsequently preserved based on inshoe plantar pressure measurement and analysis **Control (C):** Custom‐made footwear that did not undergo improvement based on in‐shoe pressure measurement **Follow‐up period:** 3 months	**Numbers**: 171 I: 85 C: 86 **Age:** I: 62.6 ± 10.2 C: 63.9 ± 10.1 **Males:** I: 82.3% C: 82.6% **Other characteristics:** **Type 2 DM:** I: 67.1% C: 75.6% **Diabetes duration (years):** I: 19.9 ± 15.1 C: 14.7 ± 11.2 **Caucasian:** I: 97.6% C: 93% **HbA1C:** I: 7.5 ± 1.4 C: 7.6 ± 1.5 **Loss of protective sensation:** I: 94.1% C: 91.9% **Vibration perception threshold >** **25 V:** I: 85.2% C: 85.9% **Peripheral arterial disease:** I: 28.8% C: 37.5% **Foot deformity:** I: 95.3% C: 97.7% **Peripheral arterial disease:** I: 28.8% C: 37.5% **Barefoot peak pressure at baseline (kPa)** **At the previous ulcer location:** I: 675 ± 392 C: 780 ± 396 **At the highest‐pressure location:** I: 934 ± 294 C: 1025 ± 286 **In‐shoe peak pressure at footwear delivery (kPa):** **At all regions of interest >** **200 kPa:** I: 269 ± 62 C: 273 ± 56 **Previous ulcer location >** **200 kPa:** I: 281 ± 68 C: 316 ± 87 **Previous ulcer location <** **200 kPa:** I: 124 ± 44 C: 126 ± 40	**In‐shoe peak pressure at footwear delivery (kPa):** **At all regions of interest >** **200 kPa** I: 221 ± 51 C: 274 ± 66 *p* = < 0.001 **Previous ulcer location** **>** **200 kPa** I: 200 ± 47 C: 304 ± 101 *p* = < 0.001 **Previous ulcer location <** **200 kPa** I: 127 ± 44 C: 133 ± 42 *p* = 0.17 **Daily step count** I: 7287 ± 3738 C: 6171 ± 3175 *p* = 0.045 **Adherence** I: 70.2 ± 25.0 C: 75.5 ± 23.4 *p* = 0.18 **Ulcer recurrence:** **Patients with ulcer:** I: 38.8% C: 44.2% *p* = 0.48 **At previous ulcer location:** I: 57.6% C: 63.2% *p* = 0.63 **Complicated foot ulcers:** I: 0% C: 16.2% *p* = 0.63 **Adherent patients:** I: 35% C: 44% **Adherent patients with ulcer:** I: 25.7% C: 47.8% *p* = 0.045 **Non‐adherent patients:** I: 39% C: 22% **Non‐adherent patients with ulcer:** I: 41% C: 34.4% *p* = 0.57 **Patients with a nonulcerative lesion:** I: 36.5% C: 45.3% *p* = 0.24 **Nonulcerative lesions** I: 76% C: 83%	**Custom‐made footwear without pressure‐based improvements**
**Collings et al. (2023)** **Intervention categories:** Insole **Outcomes reported:** Mean peak plantar pressure (MPPP). Foot ulceration	**Study design:** RCT **Setting(s):** UK.	**Population:** Participants were aged 18 years and with type 1 or type 2 diabetes and sensory DPN. **Exclusion criteria:** Non‐healing foot ulcer at another site on the plantar aspect of the foot, Gross foot deformity, e.g., Charcot foot or fixed rearfoot deformity, Major amputation **Intervention (I):** Instant optimized insole **Control (C):** Cushioned inlay insole **Follow‐up period:** 3, 6, 12 months	Numbers: 31 I: 30 C: 31 **Age:** I: 70.2 ± 10.2 C: 67.9 ± 12.2 **Males:** I: 90% C: 83.9% **BMI:** I: 30.4 ± 5.5 C: 30.4 ± 4.6 **Other characteristics:** **Type 2 DM:** I: 93.3% C: 77.4% **Diabetes duration (years):** I: 19.7 ± 14.9 C: 21.3 ± 9.7 **Ethnicity (white):** I: 100% C: 100% **Smoker:** I: 6.7% C: 9.7% **Previous foot ulceration:** I: 50% C: 51.6%	**Active insole mean MPPP kPa** **At all regions of interest >** **200 kPa** Baseline: C: 564 ± 223 I: 583.3 ± 220.9 Immediately post‐randomization: C: 447.4 ± 181.9 I: 370.2 ± 162.1 3‐month follow‐up: C: 546.1 ± 229.6 I: 495.9 ± 244.4 6‐month follow‐up: C: 639.6 ± 332.3 I: 625.3 ± 353.8 12‐month follow‐up: C: 854.7 ± 538.9 I: 596.2 ± 437.6 **Ulcer recurrence:** **Patients with ulcer:** I: 33.2% C: 22.5%	**Cushioned inlay insoles in addition to usual care; routine podiatric care**
**Farhat et al. (2024)** **Intervention categories:** Insole **Outcomes reported:** pressure points. foot ulceration. foot functionality	**Study design:** RCT **Setting(s):** UK.	**Population:** Participants over 18 years of age, of both sexes, with DM 2, with typical diabetic neuropathy. **Exclusion criteria:** Movement disorders a previous history of stroke, previous orthopedic surgeries ulceration amputation of the lower limbs pregnant women **Intervention (I):** customized insoles, which were equipped with a retro‐capital bar and an ethylene vinyl acetate (EVA) plate shaped as the retro‐capital bar. **Control (C):** Usual insole **Follow‐up period:** 6 months	Numbers: 61 I: 30 C: 31 **Age:** I: 60.5 ± 9.8 C: 63.2 ± 7.0 **Males:** I: 21.7% C: 21.7% **BMI:** I: 27.9 ± 4.0 C: 31.9 ± 6.9 **Other characteristics:** **SAH:** I: 43.5% C: 43.5% **HbA1c:** I: 8.8 ± 1.8 C: 8.0 ± 1.9 **Diabetes duration (years):** I: 15.2 ± 11.2 C: 15.3 ± 9.7 **Neuropathy duration (years):** I: 4.8 ± 6.4 C: 3.5 ± 3.3 **Drinker:** I: 8.7% C: 10.9% **Smoker:** I: 4.3% C: 6.5% **Schooling:** **Elementary school:** I: 23.9% C: 13.0% **High school:** I: 19.6% C: 15.2% **Higher education:** I: 2.2% C: 13.0% **Illiterate:** I: 8.7% C: 4.3% **Previous foot ulceration:** I: 50% C: 51.6% **Dyslipidemia:** I: 28.3% C: 32.6% **Sedentarism:** I: 32.6% C: 30.4% **Deformity:** I: 32.6% C: 23.9%	**Motor assessment:** **Right plantar flexion** C: 4.4 ± 0.8 I: 4.4 ± 1.0 *p* = 0.888 **Right Dorsal flexion** C: 4.1 ± 1.3 I: 4.2 ± 1.3 *p* = 0.776 **Right Hallux extension** C: 4.3 ± 1.1 I: 4.0 ± 1.4 *p* = 0.471 **Left plantar flexion** C: 4.4 ± 0.9 I: 4.5 ± 0.8 *p* = 0.785 **Left Dorsal flexion** C: 4.1 ± 1.3 I: 4.0 ± 1.3 *p* = 0.670 **Left Hallux extension** C: 4.3 ± 1.0 I: 3.8 ± 1.5 *p* = 0.242 **Right Ankle Jerk Reflex:** **Not evaluated:** C: 2.2% I: 0% **1+:** C: 10.9% I: 10.9% **2+:** C: 15.2% I: 15.2% **3+:** C: 2.2% I: 0% **4+:** C: 19.6% I: 23.9% *p* = 0.699 **Left Ankle Jerk Reflex:** **Not evaluated:** C: 2.2% I: 0% **1+:** C: 13.0% I: 15.2% **2+:** C: 15.2% I: 6.5% **3+:** C: 2.2% I: 2.2% **4+:** C: 17.4% I: 26.1% *p* = 0.481	**Right Tactile Sham insoles (non‐customized) or standard insoles without structural alteration** **sensitivity evaluation:** **0:** C: 8.7% I: 8.7% **1:** C: 6.5% I: 4.3% **2:** C: 2.2% I: 6.5% **3:** C: 2.2% I: 8.7% **4:** C: 4.3% I: 2.2% **5:** C: 0% I: 2.2% **6:** C: 6.1% I: 17.4% *p* = 0.527 **Left Tactile sensitivity evaluation:** **0:** C: 10.9% I: 0% **1:** C: 6.5% I: 4.3% **2:** C: 0% I: 64.3% **3:** C: 6.5% I: 4.3% **4:** C: 4.3% I: 8.7% **5:** C: 0% I: 10.9% **6:** C: 21.7% I: 17.4% *p* = 0.039 **Right Vibratory sensitivity:** **0:** C: 37.8% I: 35.6% **1:** C: 11.1% I: 15.6% *p* = 0.738 **Left Vibratory sensitivity:** **0:** C: 31.1% I: 33.3% **1:** C: 17.8% I: 17.8% *p* = 0.579
**López‐Moral et al. (2018)** **Intervention categories:** footwear with a rigid rocker sole **Outcomes reported:** Ulcer recurrence.	**Study design:** RCT **Setting(s):** Spain.	**Population:** Participants with type 1 or type 2 diabetes, age > 18 years, loss of protective foot sensation and previous foot ulcer under. **Exclusion criteria:** history of rheumatoid disease a previous history of stroke other causes of neuropathy ulceration amputation critical limb ischemia Charcot foot **Intervention (I):** Therapeutic footwear with high toe box; enough width to accommodate toe deformities and rigid rocker sole **Control (C):** Therapeutic footwear with high toe box; enough width to accommodate toe deformities and rigid sole **Follow‐up period:** 6 months	Numbers: 51 I: 26 C: 25 **Age:** I: 61 ± 8.1 C: 60 ± 8.6 **Males:** I: 92% C: 92% **BMI:** I: 28.7 ± 4.9 C: 30 ± 4.2 **Other characteristics:** **T2D:** I: 100% C: 96% **HbA1c:** I: 7.5 ± 1.7 C: 7.5 ± 1.9 **Diabetes duration (years):** I: 14 ± 8.4 C: 17 ± 10 **Retinopathy:** I: 50% C: 38% **Neuropathy:** I: 8% C: 12% **Previous amputation:** I: 50% C: 36% **Ankle mobility joint:** I: 89.5 ± 5.5 C: 88.1 ± 4.5 **Foot deformity:** I: 85% C: 72% **PAD:** I: 19% C: 32% **Foot posture index:** I: 1.6 ± 4.8 C: 2.7 ± 5.2 **Hallux mobility joint:** I: 28.6 ± 18.5 C: 29.4 ± 19.0	**Ulceration incidence:** I: 23% C: 64%	**Therapeutic footwear with semi‐rigid sole; routine care**
**Lavery et al. (2012)** **Intervention categories:** shear‐reducing insole **Outcomes reported:** Ulcer recurrence.	**Study design:** RCT **Setting(s):** USA.	**Population:** Participants with type 1 or type 2 diabetes, age > 18 years, history of a foot ulceration, or presence of sensory neuropathy with loss of protective sensation and foot deformity. **Exclusion criteria:** severe peripheral vascular disease active foot infection Dementia, history of drug or alcohol abuse amputation Charcot foot **Intervention (I):** Therapeutic footwear with shear‐reducing insole **Control (C):** Therapeutic footwear with usual insole **Follow‐up period:** 18 months	Numbers: 299 I: 149 C: 150 **Age:** I: 69.4 ± 10.0 C: 71.5 ± 7.9 **Males:** I: 68.5% C: 66.7% **Other characteristics:** **Diabetes duration (years):** I: 13 ± 8.7 C: 12 ± 4.9 **Amputation history:** I: 12.0% C: 8.6% **Ulcer history:** I: 27.5% C: 25.3% **Previous amputation:** I: 50% C: 36% **Right ankle brachial index:** I: 0.97 ± 0.11 C: 0.98 ± 0.13 **Left ankle brachial index:** I: 0.95 ± 0.11 C: 0.99 ± 0.12 **Sensory neuropathy:** I: 100% C: 100% **Right Semmes Weinstein monofilament:** I: 5.7 ± 2.7 C: 5.1 ± 2.5 **Left Semmes Weinstein monofilament:** I: 6.3 ± 2.8 C: 4.4 ± 2.2 **Right vibration perception threshold:** I: 28.2 ± 17.3 C: 30.0 ± 16.1 **Left vibration perception threshold:** I: 29.6 ± 14.8 C: 28.0 ± 14.0 **Right ankle equinus:** I: 43.6% C: 56.4% **Left ankle equinus:** I: 65.3% C: 53.7% **Hallux rigidus, right:** I: 51.0% C: 58.4% **Hallux rigidus, left:** I: 51.0% C: 58.4% **Hallux mobility joint:** I: 28.6 ± 18.5 C: 29.4 ± 19.0	**Ulceration incidence:** I: 2% C: 6.6%	**Healing sandals (HS) or removable boots without shear‐reducing features; usual care**
**Reiber et al. (2002)** **Intervention categories:** Therapeutic shoes **Outcomes reported:** Foot ulceration.	**Study design:** RCT **Setting(s):** USA.	**Population:** Participants with type 1 or type 2 diabetes, age 48–81 years, history of a full thickness foot lesions or foot infections, and loss of foot deformity. **Exclusion criteria:** Presence of healed or unhealed lesions amputation Terminal illness **Intervention (I):** I1: customized medium density cork inserts with neoprene closed‐cell cover I2: prefabricated, tapered polyurethane inserts with a brushed nylon cover **Control (C):** Usual footwear **Follow‐up period:** 24 months	Numbers: 400 I1: 121 I2: 119 C: 160 **Age:** I1: 61 ± 10.1 I2: 62 ± 10.1 C: 63 ± 10.0 **Males:** I1: 78% I2: 77% C: 66.7% **Other characteristics:** **T2M:** I1: 93% I2: 95% C: 92% **BMI:** I1: 33 ± 6.8 I2: 32 ± 6.9 C: 33 ± 7.2 **Education (years):** I1: 15 ± 2.8 I2: 14 ± 2.4 C: 14 ± 3.5 **Married:** I1: 66% I2: 62% C: 57% **Employed:** I1: 37% I2: 33% C: 28% **Ethnicity (white):** I1: 79% I2: 82% C: 74% **Foot finding:** **Moderate/severe edema:** I1: 11% I2: 8% C: 11% **No palpable pulses:** I1: 1% I2: 1% C: 2% **Insensate to monofilament:** I1: 59% I2: 66% C: 52% **Moderate foot deformity:** I1: 36% I2: 22% C: 35%	**Number of ulcers:** I1: 26% I2: 31% C: 38% **Number of nonulcerative lesions:** I1: 165% I2: 141% C: 176% **Incidence per person:** I1: 18% I2: 17% C: 27% **Incidence per person‐year:** I1: 26% I2: 31% C: 38% **Ulcer episodes:** I1: 25% I2: 22% C: 37%	**Usual footwear without therapeutic modification; routine care**
**Rizzo et al. (2012)** **Intervention categories:** Custom‐made Orthesis and shoes **Outcomes reported:** Foot ulceration.	**Study design:** RCT **Setting(s):** Italy.	**Population:** Participants with known duration of diabetes of at least 5 years, and 18 years or older. **Exclusion criteria:** active or recent ulcers active Charcot's foot local ischemia Inability of standing or walking without help, life expectancy less than 1 year **Intervention (I):** Orthesis and shoes manufactured according to the indications of the algorithm published by Dahmen et al. **Control (C):** Usual footwear **Follow‐up period:** 12, 36, 60 months	Numbers: 298 I: 149 C: 150 **Age:** I: 68.1 ± 14.1 C: 66.2 ± 9.4 **Males:** I: 68.5% C: 66.7% **Other characteristics:** **T2D:** I: 85.8% C: 82% **Diabetes duration (years):** I: 18.1 ± 12.1 C: 17.4 ± 12.9 **HbA1c:** I: 8.6 ± 1.4 C: 8.7 ± 1.1 **vibration perception threshold:** I: 27.6 ± 6.1 C: 26.1 ± 5.2	**Patients developing ulcers:** I: 17% C: 58% *p* = < 0.0001 **Number of new ulcers:** I: 20% C: 75% *p* = < 0.0001	**Standard footwear with a prevention program; routine care**
**Scirè et al. (2009)** **Intervention categories:** Digital off‐loading silicone padding **Outcomes reported:** Foot ulceration. number of areas of hyperkeratosis hardness of the skin adverse events	**Study design:** RCT **Setting(s):** Italy.	**Population:** Participants with older than 18 years, diagnosis with type 1 or type 2 diabetes mellitus for at least 5 years, peripheral neuropathy. **Exclusion criteria:** active or recent ulcers peripheral macroangiopathy erythema, edema, increase in temperature, secretions, skin macerations, pain, or tenderness fever, leukocytosis Infection clinically visible rhagades or dyshidrosis Charcot's neuroarthropathy presence of peripheral neuropathies **Intervention (I):** A digital orthosis was made to measure; Podikon 10 and 22 shores with silicone **Control (C):** Usual footwear **Follow‐up period:** 3 months	Numbers: 177 I: 89 C: 78 **Age:** I: 58.2 ± 17.1 C: 54.9 ± 18.2 **Other characteristics:** **T2D:** I: 86.5% C: 89.7% **Diabetes duration (years):** I: 15.2 ± 8.9 C: 16.4 ± 9.4 **HbA1c:** I: 8.2 ± 1.7 C: 7.9 ± 0.9 **Vibration perception threshold:** I: 37.4 ± 10.2 C: 34.1 ± 9.9 **Deformity:** I: 6% C: 8% **Hyperkeratosis:** I: 5% C: 6% **Deformity and hyperkeratosis:** I: 89% C: 86%	**Hardness of skin:** I: 71.7 ± 12.4 C: 69.8 ± 16.1 *p* = 0.42 **Prevalence of hyperkeratosis:** I: 41% C: 84% *p* = 0.002 **Ulcer incidence:** I: 1.1% C: 15.4% *p* < 0.001	**Standard therapy without silicone padding; routine care**
**Uccioli et al. (1995)** **Intervention categories:** Shoes **Outcomes reported:** Foot ulceration.	**Study design:** RCT **Setting(s):** Italy.	**Population:** Participants with older than 18 years, diagnosis with type 1 or type 2 diabetes mellitus. **Exclusion criteria:** active or recent ulcers amputation foot deformity Charcot's joints **Intervention (I):** Therapeutic shoes with custom‐molded insoles **Control (C):** Usual shoes **Follow‐up period:** 12 months	Numbers: 69 I: 33 C: 36 **Males:** I: 60.6% C: 63.8% **Age:** I: 59.6 ± 11 C: 60.2 ± 8.2 **Other characteristics:** **T2D:** I: 75.5% C: 75% **Diabetes duration (years):** I: 16.8 ± 12.7 C: 17.5 ± 8 **HbA1c:** I: 8.2 ± 1.7 C: 7.9 ± 0.9 **Vibration perception threshold:** I: 33 ± 9 C: 31 ± 12 **Ankle/brachial index:** I: 0.95 ± 0.2 C: 1 ± 0.2	**Hardness of skin:** I: 71.7 ± 12.4 C: 69.8 ± 16.1 *p* = 0.42 **Prevalence of hyperkeratosis:** I: 41% C: 84% *p* = 0.002 **Ulcer incidence:** I: 1.1% C: 15.4% *p* < 0.001	**Self‐selected shoes or non‐manufactured standard shoes; routine care**
**Ulbrecht et al. (2014)** **Intervention categories:** In‐shoe Orthoses **Outcomes reported:** Foot ulceration.	**Study design:** RCT **Setting(s):** USA.	**Population:** Participants with older than 18 years, diagnosis with type 1 or type 2 diabetes mellitus, loss of protective sensation, recently healed plantar MTH‐related foot ulcer, peak barefoot plantar pressure in the area of this previous ulcer > 450 kPa **Exclusion criteria:** Using ankle–foot orthosis existing footwear intervention **Intervention (I):** shape‐ and pressure‐based orthoses ( **Control (C):** Usual orthoses **Follow‐up period:** 15 months	Numbers: 130 I: 66 C: 64 **Males:** I: 75.8% C: 81.3% **Age:** I: 60.5 ± 10.1 C: 58.5 ± 10.7 **BMI:** I: 32.3 ± 7.1 C: 31.4 ± 5.5 **Other characteristics:** **Smoking:** I: 9.1% C: 18.8% **Ethnicity (white):** I: 83.3% C: 79.7% **Socioeconomic:** **At least high school graduate:** I: 74.2% C: 82.3% **Graduate college:** I: 18.2% C: 28.1% **Not living alone:** I: 75.8% C: 73.4% **Vibration perception threshold:** I: 33 ± 9 C: 31 ± 12 **Ankle/brachial index:** I: 1.05 ± 0.16 C: 1.13 ± 0.18 **Barefoot peak plantar pressure at prior index ulcer site:** I: 946 ± 266 C: 967 ± 233 **Barefoot peak plantar pressure at any site in either foot:** I: 1109 ± 173 C: 1085 ± 191 **Prior minor amputation:** I: 31.8% C: 37.5% **Neuro quality of life:** **Physical symptoms:** I: 1.56 ± 0.85 C: 1.38 ± 0.87 **Psychological symptoms:** I: 1.57 ± 1.13 C: 1.64 ± 1.16	**Ulcer incidence:** I: 9.9% C: 25% *p* < 0.001	Standard‐of‐care orthoses manufactured on the basis of shape and clinical information alone; routine care

*Note:* The bold values (0–6) represent the categorical scores used for tactile sensitivity assessment, while the values 0 and 1 represent the categorical scores used for vibratory sensitivity assessment, as reported in the original study by Farhat et al. (2024). Ankle jerk reflex was graded using the standard deep tendon reflex scale: 1+ = diminished (hyporeflexia), 2+ = normal, 3+ = brisk, and 4+ = hyperactive (hyperreflexia). “Not evaluated” indicates that the ankle jerk reflex was not assessed.

#### 3.2.1 Narrative Synthesis of Results on Ulcer Recurrence

Ten studies with a total of 1686 participants investigated the effect of various orthoses on preventing DFU recurrence. The results are summarized narratively below and in Table 1. Nine of the ten included studies reported findings that favored the orthosis intervention group. For instance:• Rizzo et al. [22] found a significantly lower ulcer recurrence in the custom orthosis group (12.8%) compared to controls (38.6%) at 12 months (*p* < 0.0001). López‐Moral et al. [20] reported a lower recurrence rate in a group using rigid rocker‐soled shoes (23%) compared to a semi‐rigid sole group (64%). Ulbrecht et al. [25] found a significant difference in ulcer prevention at 180 days, favoring the experimental orthosis group (*p* = 0.003). One study by Reiber et al. [21] found no significant difference in reulceration rates between the therapeutic footwear and control groups. The high degree of variability in interventions (custom insoles, different types of therapeutic footwear), control conditions (often described as “usual care” or “standard footwear”), and study populations made it inappropriate to pool the results quantitatively in a meta‐analysis.

From 4166 records, 10 RCTs were included. Narrative synthesis showed that custom orthoses reduced recurrence in several studies (e.g., Bus 2013), but evidence is heterogeneous. Publication bias could not be fully assessed due to the lack of quantitative pooling.

### 3.3 Risk of Bias Assessments

#### 3.3.1 Selection Bias

**FIGURE 2:**
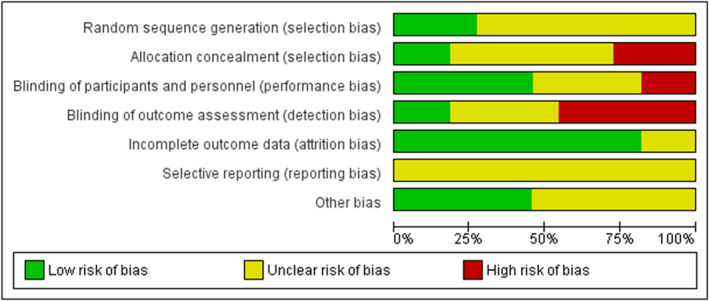
Methodological quality graph: review authors' judgments about each methodological quality item presented as percentages across all included studies.

**FIGURE 3:**
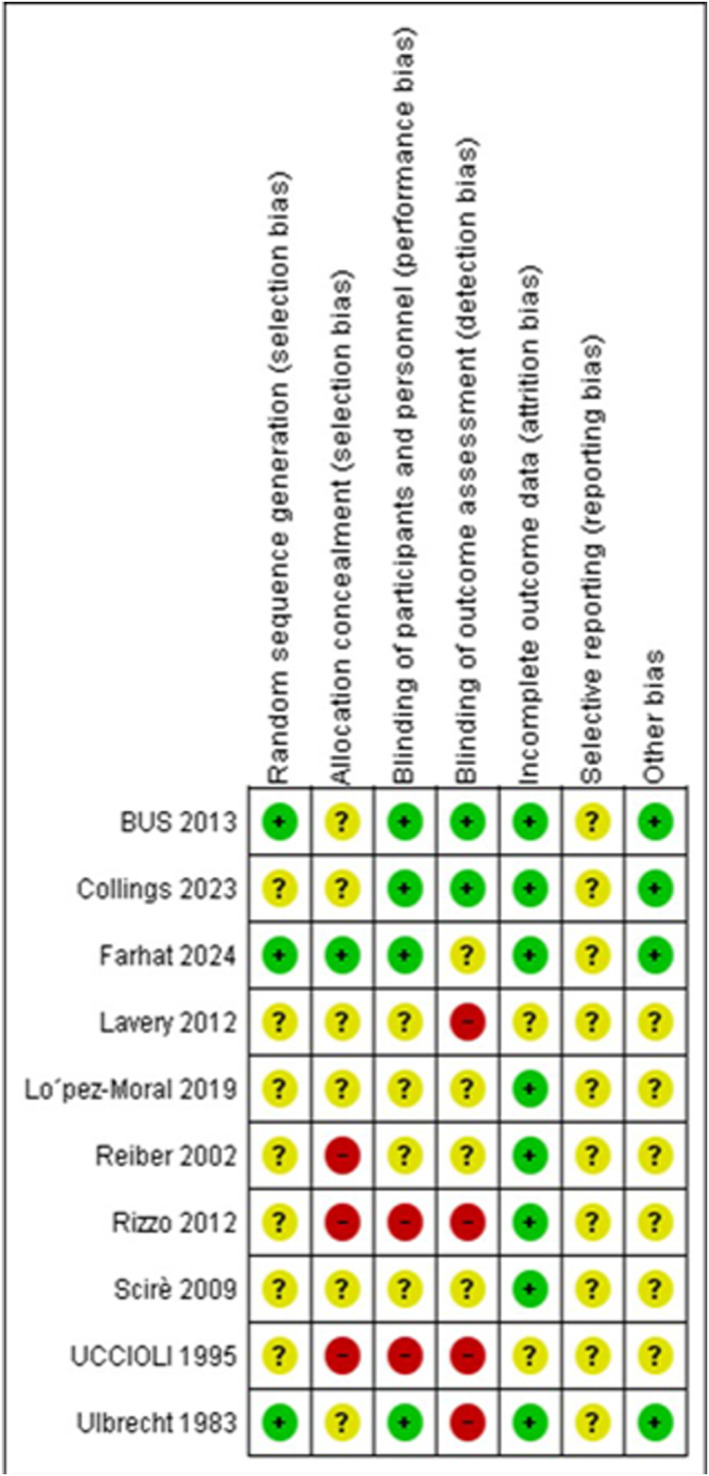
Diagram of risk of bias in the included studies.

There were seven RCTs that failed to describe their random sequence generation methods and were rated as unclearly biased [16, 18, 19, 20, 21, 22, 23]. The methodology of generating random sequences has been explained in other studies, so they are considered low‐risk studies. Allocation concealment was not specified in one study, and the bias risk was therefore considered high [20, 21, 23]. A low bias assessment has been given to other studies the use of allocation concealment (Figures 2 and 3).

#### 3.3.2 Performance Bias and Detection Bias

In four RCTs, blinding was reported, but its implementation was not described, and evidence of bias was rated unclear [18, 19, 20, 22]. In two RCTs, neither blinding of participants nor personnel was described, although blinding of outcome assessments was described. High risks were associated with both blinding outcome assessments and blinding personnel [21, 23]. In four RCTs, participants were blinded, but not measurers, so there was an unclear risk of detection bias [17, 19, 20, 22]. In terms of blinding of outcome assessment, it was rated as high risk [18, 21, 23, 24]. Other RCTs, which used blind methods or objective outcomes, were considered to have low bias because their methods were blinded to both patients and researchers (Figures 2 and 3).

#### 3.3.3 Attrition Bias and Reporting Bias

Due to the balance in the number of RCTs, and the fact that none of them had incomplete outcomes or the reason for missing outcomes, other RCTs are assessed as being low risk of bias and are rated as unclearly attributable to attrition bias. Two studies have insufficient information to determine whether there is incomplete outcome data, so they are considered at low bias risk [18, 23]. Other RCTs are considered low‐risk of bias due to the lack of incomplete outcomes or selective reporting (Figures 2 and 3).

### 3.4 Quality of Evidence

As shown in the Table 2, the results of the quality assessment of the evidence showed that a total of 10 randomized clinical trials were included in the analysis. Despite the appropriate design of most studies, the quality assessment according to the GRADE criteria indicated shortcomings in several areas. The risk of bias was assessed as serious in many studies, as in most of them the method of generating the random sequence and the method of concealing the allocation were not clearly explained. This reduced the quality of the evidence in this respect. In addition, in some studies, the implementation of blinding for participants or assessors was not properly reported, which increased the risk of performance and detection bias. In terms of heterogeneity of studies, there was a significant degree of inconsistency between the results, due to differences in the type of orthoses, the duration of follow‐up and the populations studied. This heterogeneity led to a decrease in the level of certainty of the evidence and prevented quantitative data synthesis (meta‐analysis), so that only narrative synthesis was used to interpret the results. Although most studies directly examined the effect of orthoses on the incidence of diabetic foot ulcers, the precision of the estimates was low due to small sample sizes and wide confidence intervals, and the evidence was also reduced in terms of imprecision. Overall, the overall certainty of the evidence regarding the effect of orthoses on ulcer prevention in diabetic patients was downgraded from “low” to “very low” due to high risk of bias, significant heterogeneity, and statistical imprecision. Therefore, although the results of some studies suggested a potentially positive effect of orthoses on reducing ulcer incidence, a definitive conclusion cannot be drawn in this area due to methodological limitations and insufficient evidence.

**TABLE 2:** The quality assessment of the evidence on the impact of orthoses on ulceration in diabetic patients.

Quality assessment							Certainty
No. of studies	Design	Risk of bias	Inconsistency	Indirectness	Imprecision	Publication bias	
10	Randomized trial	Serious	serious	No serious indirectness	Imprecision	undetected	Low ⊕OOO

*Note:* Downgraded to ‘Very Low’ due to serious risk of bias (e.g., unclear blinding), serious inconsistency (heterogeneous interventions), and serious imprecision (small sample sizes). Narrative synthesis was used due to clinical heterogeneity, precluding quantitative pooling.

^a^Substantial inconsistency.

^b^Considerable inconsistency.

### 3.5 Ulcer Recurrence at the End of Intervention

Ten randomized controlled trials (RCTs) with a total of 1686 participants (887 in the intervention group and 799 in the control group) evaluated the effectiveness of various orthoses in preventing diabetic foot ulcer (DFU) recurrence compared to standard care. Due to significant heterogeneity in orthosis types (e.g., custom insoles, therapeutic footwear, rocker‐soled shoes) and follow‐up periods (ranging from 3 to 18 months), a meta‐analysis was not feasible, and a narrative synthesis was conducted.

The findings consistently favored orthotic interventions in most studies. For example:

Rizzo et al. (2012) reported a significantly lower ulcer recurrence rate in the custom orthosis group (12.8%) compared to the control group receiving standard care (38.6%) at 12 months (*p* < 0.0001). This difference persisted at 3 years (17.6% vs. 61.0%, *p* < 0.0001) [21].

López‐Moral et al. (2019) found that rigid rocker‐soled shoes reduced recurrence rates (23%) compared to semi‐rigid soles (64%) in patients leaving with diabetic neuropathy [19].

Ulbrecht et al. (2014) observed a significant reduction in ulcer recurrence at 180 days with plantar pressure‐based in‐shoe orthoses compared to standard care (*p* = 0.003) [24].

Bus et al. (2013) reported a recurrence rate of 9.9% in the custom footwear group versus 25% in the control group over 18 months [15].

However, one study by Reiber et al. (2002) found no significant difference in recurrence rates between therapeutic footwear and standard care, potentially due to differences in intervention design or adherence [20]. Variability in study populations, orthosis materials, and control group definitions (e.g., standard footwear or routine education) limited direct comparisons across studies. These findings suggest that custom‐made orthoses, particularly those designed to offload pressure, are generally effective in reducing DFU recurrence, though results vary based on intervention type and study duration. Further details on individual study outcomes are provided in Table 1.

## 4 Discussion

This systematic review aimed to evaluate the effectiveness of various orthoses in preventing the recurrence of DFUs. DFUs are a major complication of diabetes, often leading to amputations, hospitalizations, and reduced quality of life [25]. Recurrence is commonly driven by excessive mechanical stress on insensitive feet, caused by plantar pressure, shear stress, and repetitive ambulatory activity [7]. Such stress increases the risk of ulceration and subsequent complications, including infection and amputation [26]. Orthotic interventions, such as custom‐made insoles, therapeutic footwear, and rocker‐soled shoes, are designed to reduce mechanical stress and prevent DFU recurrence. This review successfully achieved its objective of assessing the effectiveness of orthotic devices in reducing DFU recurrence rates compared to standard care.

Narrative synthesis of 10 randomized controlled trials (RCTs) involving 1686 participants demonstrated that custom‐made orthoses significantly reduced DFU recurrence in most studies. For instance, Rizzo et al. (2012) found a significantly lower recurrence rate in the custom orthosis group (12.8%) compared to the control group (38.6%) at 12 months (*p* < 0.0001), with benefits persisting at 3 years (17.6% vs. 61.0%, *p* < 0.0001) [21]. Similarly, López‐Moral et al. (2019) reported that rigid rocker‐soled shoes reduced recurrence rates to 23% compared to 64% in the semi‐rigid sole group among individuals with diabetic neuropathy [19]. Ulbrecht et al. (2014) observed a significant reduction in recurrence at 180 days with plantar pressure‐based in‐shoe orthoses (*p* = 0.003) [24]. Bus et al. (2013) reported a recurrence rate of 9.9% in the custom footwear group versus 25% in controls over 18 months [15]. However, Reiber et al. (2002) found no significant difference in recurrence rates between therapeutic footwear and standard care, possibly due to variations in intervention design or adherence [20].

The findings align with prior research emphasizing the role of pressure offloading in preventing DFU recurrence. Custom‐made orthoses outperform off‐the‐shelf options due to their tailored fit and ability to address individual pressure distribution needs, thereby reducing recurrence rates. Unlike previous reviews that focused on specific offloading devices, this study comprehensively evaluates various orthoses and their impact on preventing DFU recurrence [25, 27, 28, 29, 30, 31]. The inclusion of recent studies on custom‐made orthoses strengthens the evidence base.

Despite these findings, limitations exist. Variability in study design, orthosis materials, and follow‐up periods (3–18 months) introduced heterogeneity, complicating direct comparisons. The lack of standardized definitions for “standard care” across studies further limits generalizability. Additionally, the higher cost and production time of custom orthoses compared to off‐the‐shelf options pose challenges for widespread clinical adoption. Future research should standardize methodologies, define control conditions clearly, and explore cost‐effective orthotic solutions to enhance accessibility and applicability in clinical practice.

## 5 Conclusions

In conclusion, this systematic review with narrative synthesis of randomized controlled trials highlights the varying effectiveness of different types of orthoses in preventing the recurrence of diabetic foot ulcers. The findings suggest that custom‐made orthoses may offer potential benefits in reducing recurrence rates compared to standard care, as seen in prevention‐focused studies (e.g., Bus 2013 and Ulbrecht 2014), though results varied due to heterogeneity. Further research is needed to fully understand the comparative advantages and optimal use of these interventions in clinical practice.

## Author Contributions

**Behnam Amini:** investigation; methodology; resources; software; writing – original draft. **Hanieh Salehi‐Pourmehr:** data curation; formal analysis; methodology; software; visualization; writing – original draft. **Sevil Ghaffarzadehrad:** project administration; supervision; validation; writing – review & editing.

## Funding

The authors have nothing to report.

## Conflicts of Interest

The authors declare no conflicts of interest.

## Supporting information


Supporting Information S1

## Data Availability

The data that support the findings of this study are available from the corresponding author upon reasonable request.
