# Supplementary material for: Comparing the Effectiveness of Different Types of Orthoses in Preventing Diabetic Foot Ulcer Recurrence: A Systematic Review of Randomized Clinical Trials
Source: J Foot Ankle Res. 2026 Aug 1;19(3):e70192. doi: 10.1002/jfa2.70192 (PMC13428632; doi:10.1002/jfa2.70192)
Supplement: Supplementary file 1 — Supporting Information S1 [file JFA2-19-e70192-s001.docx]

Searching strategies for databases

| **Database** | **Strategy** | **N** |
| --- | --- | --- |
| PubMed | 1= ("Foot Ulcer"[MeSH Terms] OR "Diabetic Foot"[MeSH Terms] OR "Wound Healing"[MeSH Terms] OR "Neuropathy"[Title/Abstract] OR "Ulcer Healing"[Title/Abstract] OR "Wound Healings"[Title/Abstract] OR ("Inflammatory" AND "Wound Healing"[Title/Abstract]) OR ("Fibroblast*" AND "Wound Healing"[Title/Abstract]) OR ("Maturation" AND "Wound Healing"[Title/Abstract]) OR ("Wagner" AND "Diabetic foot Ulcer"[Title/Abstract]) OR "foot disease"[Title/Abstract] OR "wound"[Title/Abstract] OR "Pressure ulcer"[Title/Abstract] OR "Plantar Ulcer"[Title/Abstract] OR "Foot Ulcer*"[Title/Abstract] OR "Diabetic Feet"[Title/Abstract] OR "Diabetic Foot*"[Title/Abstract])  2= ("Foot Orthoses"[MeSH Terms] OR "Braces"[MeSH Terms] OR "Orthotic Devices"[MeSH Terms] OR "Therapeutic Footwear"[Title/Abstract] OR "Diabetic Shoes"[Title/Abstract] OR shoes[Title/Abstract] OR boots[Title/Abstract] OR Casts[Title/Abstract] OR "Smart Shoes"[Title/Abstract] OR "Braces"[Title/Abstract] OR "Ankle-Foot Orthosis"[Title/Abstract] OR "AFO"[Title/Abstract] OR "Total Contact Cast"[Title/Abstract] OR "TCC"[Title/Abstract] OR "Foot Arch Support"[Title/Abstract] OR "footwear"[Title/Abstract] OR "Orthoses"[Title/Abstract] OR "Offloading"[Title/Abstract] OR "Orthosis"[Title/Abstract] OR "Orthotic Devices"[Title/Abstract] OR "Insoles"[Title/Abstract] OR "Custom Insoles"[Title/Abstract] OR "Prefabricated Insoles"[Title/Abstract] OR "Foot Orthosis"[Title/Abstract] OR "Foot Orthotic Device"[Title/Abstract] OR "Orthotic Insoles"[Title/Abstract] OR ("Orthotic" AND "Shoe Insert"[Title/Abstract]) OR ("Toe" AND "Box Shoes"[Title/Abstract]))  3= ("Diabetes Mellitus"[MeSH Terms] OR "Diabetes Mellitus, Type 2"[MeSH Terms] OR "Diabetes Mellitus, Type 1"[MeSH Terms] OR "Diabetes Mellitus"[Title/Abstract] OR "Diabetes, Type 2"[Title/Abstract] OR "Diabetes Mellitus, Type II"[Title/Abstract] OR "Diabetes Mellitus, Type 1"[Title/Abstract] OR "Diabetes, Type 1"[Title/Abstract] OR "Diabetes Mellitus, Type I"[Title/Abstract] OR "Type 1 Diabetes Mellitus"[Title/Abstract]) | **1+2+3= 1562** |
| WOS | **TS= ("Diabetes Mellitus" OR "Diabetes Mellitus, Type 2" OR "Diabetes Mellitus, Type 1" OR "Diabetes, Type 2" OR "Diabetes Mellitus, Type II" OR "Diabetes, Type 1" OR "Type 1 Diabetes Mellitus")**  **AND**  **TS= ("Foot Orthoses" OR "Braces"OR "Orthotic Devices"OR "Therapeutic Footwear" OR "Diabetic Shoes"OR shoes OR boots OR Casts OR "Smart Shoes" OR "Braces" OR "Ankle-Foot Orthosis" OR "AFO"OR "Total Contact Cast"OR "TCC" OR "Foot Arch Support" OR "footwear"OR "Orthoses" OR "Offloading"OR "Orthosis"OR "Orthotic Devices"OR "Insoles" OR "Custom Insoles"OR "Prefabricated Insoles" OR "Foot Orthosis" OR "Foot Orthotic Device" OR "Orthotic Insoles" OR ("Toe" AND "Box Shoes"))**  **AND**  **TS= ("Foot Ulcer*" OR "Diabetic Foot*" OR "Wound Healing" OR "Neuropathy"OR "Ulcer Healing" OR "Wound Healings" OR ("Inflammatory" AND "Wound Healing") OR ("Fibroblast*" AND "Wound Healing") OR ("Maturation" AND "Wound Healing") OR ("Wagner" AND "Diabetic foot Ulcer") OR "foot disease"OR "wound" OR "Pressure ulcer" OR "Plantar Ulcer")** | **4**39 |
| Scopus | TITLE-ABS-KEY("Diabetes Mellitus" OR "Diabetes Mellitus, Type 2" OR "Diabetes Mellitus, Type 1" OR "Diabetes, Type 2" OR "Diabetes Mellitus, Type II" OR "Diabetes, Type 1" OR "Type 1 Diabetes Mellitus") AND TITLE-ABS-KEY("Foot Orthoses" OR "Braces"OR "Orthotic Devices"OR "Therapeutic Footwear" OR "Diabetic Shoes"OR shoes OR boots OR Casts OR "Smart Shoes" OR "Braces" OR "Ankle-Foot Orthosis" OR "AFO"OR "Total Contact Cast"OR "TCC" OR "Foot Arch Support" OR "footwear"OR "Orthoses" OR "Offloading"OR "Orthosis"OR "Orthotic Devices"OR "Insoles" OR "Custom Insoles"OR "Prefabricated Insoles" OR "Foot Orthosis" OR "Foot Orthotic Device" OR "Orthotic Insoles" OR ("Toe" AND "Box Shoes")) AND TITLE-ABS-KEY("Foot Ulcer*" OR "Diabetic Foot*" OR "Wound Healing" OR "Neuropathy"OR "Ulcer Healing" OR "Wound Healings" OR ("Inflammatory" AND "Wound Healing") OR ("Fibroblast*" AND "Wound Healing") OR ("Maturation" AND "Wound Healing") OR ("Wagner" AND "Diabetic foot Ulcer") OR "foot disease"OR "wound" OR "Pressure ulcer" OR "Plantar Ulcer") | **1742** |
| Cochrane/  Cochrane Protocols and Trials | ("Diabetes Mellitus") AND ("Foot Orthoses" OR "footwear" OR "Braces" OR "Diabetic Shoes" OR "Orthotic Devices") AND ("Diabetic Feet" OR "Foot Ulcer" OR "Wound Healing" OR "foot disease" OR "Plantar Ulcer") | **74** |
| ProQuest | ABSTRACT("Foot Ulcer*" OR "Diabetic Foot*" OR "Wound Healing" OR "Neuropathy"OR "Ulcer Healing" OR "Wound Healings" OR ("Inflammatory" AND "Wound Healing") OR ("Fibroblast*" AND "Wound Healing") OR ("Maturation" AND "Wound Healing") OR ("Wagner" AND "Diabetic foot Ulcer") OR "foot disease"OR "wound" OR "Pressure ulcer" OR "Plantar Ulcer") OR TITLE ("Foot Ulcer*" OR "Diabetic Foot*" OR "Wound Healing" OR "Neuropathy"OR "Ulcer Healing" OR "Wound Healings" OR ("Inflammatory" AND "Wound Healing") OR ("Fibroblast*" AND "Wound Healing") OR ("Maturation" AND "Wound Healing") OR ("Wagner" AND "Diabetic foot Ulcer") OR "foot disease"OR "wound" OR "Pressure ulcer" OR "Plantar Ulcer")  AND ABSTRACT("Foot Orthoses" OR "Braces"OR "Orthotic Devices"OR "Therapeutic Footwear" OR "Diabetic Shoes"OR shoes OR boots OR Casts OR "Smart Shoes" OR "Braces" OR "Ankle-Foot Orthosis" OR "AFO"OR "Total Contact Cast"OR "TCC" OR "Foot Arch Support" OR "footwear"OR "Orthoses" OR "Offloading"OR "Orthosis"OR "Orthotic Devices"OR "Insoles" OR "Custom Insoles"OR "Prefabricated Insoles" OR "Foot Orthosis" OR "Foot Orthotic Device" OR "Orthotic Insoles" OR ("Toe" AND "Box Shoes")) OR TITLE ("Foot Orthoses" OR "Braces"OR "Orthotic Devices"OR "Therapeutic Footwear" OR "Diabetic Shoes"OR shoes OR boots OR Casts OR "Smart Shoes" OR "Braces" OR "Ankle-Foot Orthosis" OR "AFO"OR "Total Contact Cast"OR "TCC" OR "Foot Arch Support" OR "footwear"OR "Orthoses" OR "Offloading"OR "Orthosis"OR "Orthotic Devices"OR "Insoles" OR "Custom Insoles"OR "Prefabricated Insoles" OR "Foot Orthosis" OR "Foot Orthotic Device" OR "Orthotic Insoles" OR ("Toe" AND "Box Shoes"))  AND  [ABSTRACT("Diabetes Mellitus" OR "Diabetes Mellitus, Type 2" OR "Diabetes Mellitus, Type 1" OR "Diabetes, Type 2" OR "Diabetes Mellitus, Type II" OR "Diabetes, Type 1" OR "Type 1 Diabetes Mellitus" ) OR TITLE ("Diabetes Mellitus" OR "Diabetes Mellitus, Type 2" OR "Diabetes Mellitus, Type 1" OR "Diabetes, Type 2" OR "Diabetes Mellitus, Type II" OR "Diabetes, Type 1" OR "Type 1 Diabetes Mellitus")](https://www.proquest.com/recentsearches.recentsearchtabview.recentsearchesgridview.scrolledrecentsearchlist.checkdbssearchlink:rerunsearch/5235D69E72C4A28PQ/None/$N?t:ac=RecentSearches) | **45** |
